# Obstetric and Neonatal Outcomes following COVID-19 Vaccination in Pregnancy

**DOI:** 10.3390/jcm11092540

**Published:** 2022-04-30

**Authors:** Ravit Peretz-Machluf, Galit Hirsh-Yechezkel, Inna Zaslavsky-Paltiel, Adel Farhi, Nir Avisar, Liat Lerner-Geva, Raanan Meyer, Abraham Tsur, Yoav Yinon

**Affiliations:** 1 The Sackler Faculty of Medicine, Tel-Aviv University, Tel Aviv 6997801, Israel; ravit.machluf@gmail.com (R.P.-M.); niravisar2192@gmail.com (N.A.); liatl@gertner.health.gov.il (L.L.-G.); raananmeir@gmail.com (R.M.); avi.tsur@sheba.health.gov.il (A.T.); 2 The Department of Obstetrics and Gynecology, Chaim Sheba Medical Center, Ramat Gan 5262000, Israel; 3 Women and Children’s Health Research Unit, Gertner Institute for Epidemiology & Health Policy Research Ltd., Tel Hashomer, Ramat Gan 5266202, Israel; galith@gertner.health.gov.il (G.H.-Y.); innaz@gertner.health.gov.il (I.Z.-P.); dollyf@gertner.health.gov.il (A.F.); 4 School of Public Health, Sackler Faculty of Medicine, Tel Aviv University, Tel Aviv 6997801, Israel

**Keywords:** obstetric outcome, neonatal outcome, BNT162b2 vaccine, COVID-19, pregnancy, prenatal vaccination, meconium

## Abstract

COVID-19 infection imposes a risk for pregnant individuals and may lead to adverse maternal and obstetric outcomes. This is a retrospective cohort study of all women giving birth between March and July 2021 at a single tertiary center. Obstetric and neonatal outcomes were compared between vaccinated and non-vaccinated pregnant women with singleton pregnancies. Women with prior COVID-19 infection, multiple gestations and stillbirth were excluded from the study. Of 4708 women who delivered during the study period, 3700 met the eligibility criteria, of whom 3240 were vaccinated during pregnancy. Compared with the non-vaccinated group, the vaccinated group was characterized by a lower rate of smoking (3.70% vs. 6.67%, *p* = 0.0028), whereasother maternal characteristics were not significantly different. Multivariable analysis demonstrated that COVID-19 mRNA vaccination was not significantly associated with increased risk of preterm birth as well as other adverse obstetric outcomes including hypertensive diseases of pregnancy, cesarean delivery and small for gestational age. However, a significantly lower risk for meconium-stained amniotic fluid was observed among the vaccinated group (adjusted odds ratio 0.63; 95% confidence interval, 0.46–0.86, *p* = 0.0039). Moreover, the vaccine was not significantly associated with increased risk of neonatal adverse outcomes including respiratory complications and NICU hospitalization. In conclusion, BNT162b2 messenger RNA vaccination during pregnancy was not associated with an increased rate of adverse obstetric and neonatal outcomes. Therefore, in view of its safety on one hand, and the risk associated with COVID-19 disease in pregnancy on the other hand, BNT 162b2 COVID-19 vaccine should be recommended for pregnant women.

## 1. Introduction

The coronavirus disease 2019 (COVID-19) pandemic has led to substantial morbidity and mortality globally during the last two years [1]. Accumulating evidence indicates that pregnant women are more likely to experience COVID-19 complications compared with non-pregnant women, including need for invasive ventilation, admission to an intensive care unit, and death. Moreover, COVID-19 in pregnancy has also been shown to be associated with increased risk for preterm birth, preeclampsia, and stillbirth [2,3,4]. 

Since approved by the Food and Drug Administration [5], the mRNA-based vaccines were found safe and highly effective for preventing symptomatic COVID-19 disease in the general population aged 16 and older [6,7]. However, pregnant women were excluded from all initial clinical trials reviewing BNT162b2 vaccine. Nevertheless, due to the risk imposed and the promising observational studies, the CDC [8], ACOG [9], and the Israeli ministry of health [10] have each published recommendations supporting vaccination during pregnancy and lactation. Recent observational studies have shown that mRNA vaccination during pregnancy seems safe and effective, similar to the general population. [11,12,13,14,15]. Additionally, studies have shown that the risk for spontaneous abortions after receiving mRNA vaccine was not elevated [16,17,18]. Furthermore, the maternal immunization response to the vaccine during pregnancy facilitated effective transfer of SARS-CoV-2 immunoglobulin G across the placenta, providing neonatal humoral immunity against COVID-19 [19,20]. However, data regarding the safety of the vaccine with respect to obstetric and neonatal outcomes is limited [15,21].

## 2. Materials and Methods

This was a retrospective cohort study including all women who delivered between March to July 2021 in a single tertiary medical center at gestational age of at least 22 weeks. Exclusion criteria included prior COVID-19 infection or unknown vaccination status, multiple gestations, stillbirth (including termination of pregnancy and intrauterine fetal death) and early neonatal death within 24 h after birth. Demographic, clinical and laboratory data were obtained using the MDClone platform and rechecked after data decryption using electronic medical records. Maternal characteristics, obstetric and short-term neonatal outcomes were compared between non-vaccinated women to women who were vaccinated by at least one dose of BNT162b2 (Pfizer/BioNTech) vaccine during pregnancy.

The statistical inquiry of the data initiated with univariate analyses for testing the differences between vaccinated and non-vaccinated women as well as the differences for each outcome variable were performed using the chi-square test and Fisher’s exact test for categorical variables and by two-sample T-test and Wilcoxon Rank Sum Test for continuous variables. All tests were two-tailed and *p*-values < 0.05 were considered statistically significant.

For each outcome variable, multivariable logistic or multinomial regression analyses were used for testing the differences between vaccinated and non-vaccinated women, with adjustment for confounding variables. 

Models for obstetric and neonatal outcomes were adjusted for maternal age, parity, BMI prior to birth, gestation age, smoking, hypertensive disorders, and diabetes prior and/or during the pregnancy (depending on the model).

Adjusted Odds Ratios (OR) are presented with 95% confidence intervals (CI). Statistical analyses were performed using SAS statistical software version 9.4 (SAS Institute, Inc., Cary, NC, USA).

## 3. Results

**Figure 1:**
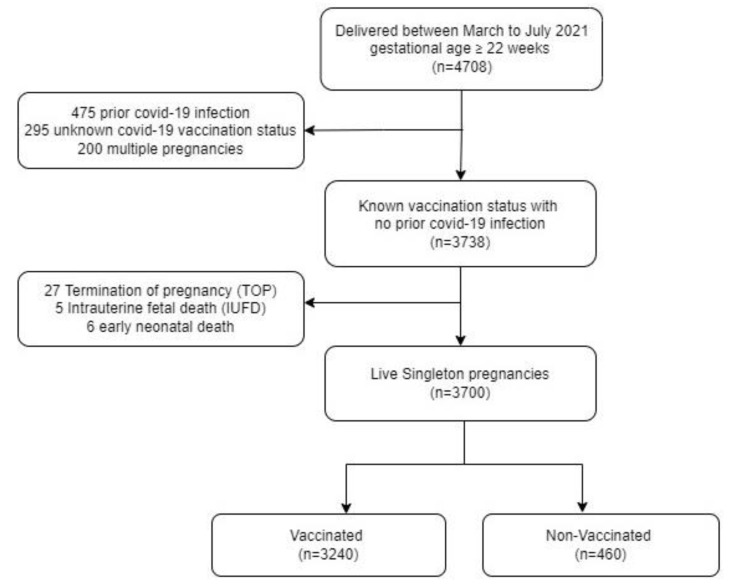
Cohort flow diagram. Inclusion criteria were as follows: (1) Women giving birth between March 2021–July 2021 with gestational age of at least 22 weeks; (2) Women with known vaccination status and no COVID-19 infection prior to giving birth; (3) Singleton live births.

From 1 March to 31 July 2021, a total of 4708 women gave birth at Sheba Medical Center, of whom 475 had prior COVID-19 infection and 295 lacked vaccination status data. A total of 200 multiple gestation pregnancies, 27 terminations of pregnancy, 5 intrauterine fetal deaths and 6 early neonatal deaths were excluded from the study (Figure 1). The final study population included 3700 live singleton deliveries. Of them, 3240 were vaccinated with BNT162b2 messenger RNA vaccine at least once during their pregnancy, and 460 women were not vaccinated.

### 3.1. Maternal Characteristics

Maternal characteristics including age (32.51 ± 5.07 vs. 31.99 ± 5.51 *p* = 0.1197), gravidity, parity, mode of conception and rate of background diseases were comparable between the two groups (Table 1). However, the vaccinated group had a lower rate of smokers (3.7% vs. 6.67%, respectively, *p* = 0.0028).

### 3.2. Obstetric Outcomes

The rates of gestational diabetes, pregnancy-induced hypertension, preeclampsia, small for gestational age (SGA) and preterm premature rupture of membranes did not differ between the two groups (Table 2). The rate of early pre-term labor (<34 weeks) was lower among the vaccinated group (0.83% vs. 1.96%, *p* = 0.0216). No differences were found between the groups with respect to mode of delivery and delivery complications such as maternal fever. However, the rate of deliveries complicated by meconium-stained amniotic fluid was significantly lower in the vaccinated group (13.75% vs. 20.05%, respectively, *p* = 0.0005).

### 3.3. Neonatal Outcomes

Neonatal outcome measures including rates of Apgar < 7 at 5 min after birth (0.31% vs. 0.22%), severe acidemia measured by venous cord blood pH < 7 (0.19% vs. 0.65%), respiratory distress syndrome (0.52% vs. 0.65%), transient tachypnea of the newborn (1.23% vs. 1.74%), meconium aspiration syndrome (0.25% vs. 0.22%), mechanical ventilation (0.83% vs. 1.30%), NICU hospitalization (2.75%, 3.48%), Intracranial hemorrhage (0.03% vs. 0.22%), hypothermia (0.03% vs. 0%), and hypoglycemia (4.91% vs. 5.65%) did not differ significantly between the two groups (Table 3).

### 3.4. Multivariable Analysis

Multivariable analyses of obstetric complications (Table 4) were adjusted for maternal age, parity, gestation age, and background conditions such as obesity (BMI ≥ 30), chronic hypertension, diabetes, and smoking. Multivariable analysis demonstrated that COVID-19 mRNA vaccination was not significantly associated with increased risk of adverse obstetric outcomes including preterm birth (adjusted odds ratio 0.94; 95% confidence interval, 0.62–1.44), hypertensive diseases of pregnancy (adjusted odds ratio 1.3; 95% confidence interval, 0.70–2.42), cesarean delivery (adjusted odds ratio 0.86; 95% confidence interval 0.64–1.14 for non-urgent cesarean delivery and adjusted odds ratio 0.78; 95% confidence interval, 0.57–1.07 for urgent cesarean delivery), and SGA (adjusted odds ratio 1.01; 95% confidence interval, 0.66–1.55).However, a significantly lower risk for meconium-stained amniotic fluid was observed among the vaccinated group (adjusted odds ratio 0.63; 95% confidence interval, 0.46–0.86, *p* = 0.0039). Multivariable analyses of adverse neonatal outcomes (Table 4) were additionally adjusted to diabetes and hypertensive disorders of pregnancy. The analysis demonstrated that the vaccine was not significantly associated with increased risk of neonatal adverse outcomes including respiratory complications (adjusted odds ratio 0.74; 95% confidence interval, 0.38–1.45) and NICU hospitalization (adjusted odds ratio 1.06; 95% confidence interval, 0.54–02.1).

## 4. Discussion

In this study we evaluated the effects of Pfizer/BioNTech BNT162b2 vaccine administration during pregnancy on obstetric and neonatal outcomes for singleton pregnancies. The vaccinated and non-vaccinated groups characteristics differed only in the higher rate of smokers in the non-vaccinated group. Compared to the non-vaccinated group, no significant differences were found for all adverse outcomes analyzed except for a lower rate for meconium-stained amniotic fluid. Lower rates for early preterm birth were also observed at the vaccinated group yet were found insignificantly different when adjusted to the multivariable model. These findings, analyzed for a relatively large group of vaccinated pregnant women, contribute to the reassuring body of evidence that COVID-19 vaccine given during pregnancy is safe and does not increase the risk for adverse perinatal outcomes. 

The data accumulated so far indicate that pregnant women are at risk for developing severe COVID-19 disease, resulting in maternal and neonatal complications [2,3,4]. Recent observational studies have shown that mRNA vaccination during pregnancy seems safe and effective, similar to the general population [11,12,13,14,15]. In addition, our findings show that obstetric and neonatal outcomes following exposure during pregnancy to the BNT162b2 vaccine are comparable to those of non-vaccinated women. In accordance with our findings, a previous study on a smaller cohort of 913 vaccinated pregnant women found no adverse effect on pregnancy outcome following COVID-19 vaccination in pregnancy [22]. However, in contrast to our findings, Rottenstreich et al. have showed increased rate of elective cesarean delivery and decreased risk of adverse neonatal outcomes among 712 pregnant women who received two doses of COVID-19 vaccine during the third trimester [23].

Interestingly, we found that the risk for meconium-stained amniotic fluid was significantly lower in the vaccinated group, suggesting that the vaccine may have a protective effect against it. Meconium-stained amniotic fluid is known to be a sign of fetal distress [24], and has been reported to be more frequently found in mothers positive for SARS-CoV-2 infection [25,26]. The mRNA vaccine effect on meconium-stained amniotic fluid studied in smaller groups have shown contradicting results [22,23]. Additional studies are required to further validate whether COVID-19 mRNA vaccination is protective from meconium stained amniotic fluid.

To date, information regarding COVID-19 vaccination in pregnancy with respect to safety and perinatal outcomes is limited. Consequently, conflicting statements were published by public health groups regarding the need of pregnant women to be vaccinated creating great confusion. Our results confirm the safety of Pfizer/BioNTech BNT162b2 vaccine taking into consideration also obstetric and neonatal outcomes. In addition to the maternal protection against COVID-19, vaccination during pregnancy may also provide neonatal humoral immunity [20]. From a clinician’s perspective, in view of the risk associated with COVID-19 disease in pregnancy and the safety of the mRNA vaccine implied by this study, these findings should reassure both care givers and patients and help increase mRNA COVID-19 vaccine acceptance rates among pregnant women.

With regard to research implications, this study included all singleton live deliveries in a single tertiary center at a specific timeframe during the SARS-CoV-2 pandemic. Therefore, its population of both vaccinated and non-vaccinated pregnant women represents their equivalent exposure to the pandemic effects on prenatal care, obstetric and neonatal outcomes. This contributes to the validity of our findings, demonstrating no significant differences in adverse perinatal outcomes between the groups. However, long-term neonatal outcomes evaluation was not included in the scope of this research and requires further studies. 

Additional significant outcomes excluded from this study were births prior to 22 gestational weeks, stillbirth, and early neonatal death. Of 11 cases of stillbirths and early neonatal deaths excluded, the rates were similar between vaccinated to non-vaccinated women (0.003% vs. 0.004%, respectively, *p* = 0.5641). Furthermore, new studies have shown no increased risk for miscarriage and stillbirth in vaccinated compared to non-vaccinated pregnant women [15,17,18,21].

The main strength of this study lies within its relatively large vaccinated pregnant women group. Both obstetric and neonatal outcomes data were a product of a systematic and meticulous electronic medical records analysis that was audited in real-time and queried by trained personnel. The statistical analysis was thorough, eliminating any known clinically relevant confounders via multivariable logistic or multinomial regression tools. 

Nevertheless, this study has several limitations. First, due to a high COVID-19 infection rate in Israel at the time of the study and following the Israeli Ministry of Health recommendation, the majority of pregnant women got vaccinated, resulting in a higher rate of vaccinated pregnant women in the study population. However, as mentioned above, the relatively large volume of vaccinated pregnant women further validates the observed low rate of obstetric complications in that group. Second, most women in our cohort were vaccinated during their third trimester, restricting our conclusions from assessing the effect of vaccine exposure in earlier stages of pregnancy. Third, several potential confounders such as socio-economic status were not considered in the study analysis and may have an impact on our findings. Of note, the healthcare services in Israel including prenatal care and vaccination facilities are available to the public, highly accessible, and subsidized. Therefore, the impact of such characteristics on our findings is assumed to be low. 

Fourth, the data collected on infrequent outcomes in this study may not have met the statistical power threshold required to detect clinically relevant differences. 

## 5. Conclusions

BNT162b2 messenger RNA vaccination during pregnancy was not associated with an increased rate of adverse obstetric and neonatal outcomes. Therefore, in view of its safety on one hand, and the risk associated with COVID-19 disease in pregnancy on the other hand, BNT 162b2 COVID-19 vaccine should be recommended for pregnant women.

## Figures and Tables

**Table 1:** Maternal characteristics.

	Total ^1^	Vaccinated	Non-Vaccinated	*p*-Value
	(*n* = 3700), *n* (%)	(*n* = 3240), *n* (%)	(*n* = 460), *n* (%)	
Age ^2^		32.51 ± 5.07	31.99 ± 5.51	0.1197
Smoking	149 (4.06)	199 (3.70)	30 (6.67)	0.0028
Gravidity				0.6551
1	998 (26.99)	863 (26.65)	135 (29.35)	
2	980 (26.50)	861 (36.59)	119 (25.87)	
3	782 (21.15)	686 (21.19)	96 (20.87)	
4+	938 (25.37)	828 (25.57)	110 (23.91)	
Parity				0.1045
0	1314 (35.53)	1127 (34.81)	187 (40.65)	
1	1110 (30.02)	980 (30.27)	130 (28.26)	
2	816 (22.07)	725 (22.39)	91 (19.78)	
3+	458 (12.39)	406 (12.54)	52 (11.30)	
Mode of conception				0.5215
Spontaneous	2829 (86.54)	2477 (86.40)	352 (87.56)	
Fertility treatments	440 (13.46)	390 (13.60)	50 (12.44)	
Background conditions				
Obesity ^3^	1125 (31.73)	995 (31.97)	130 (30.02)	0.4141
Chronic Hypertension	41 (1.11)	35 (1.08)	6 (1.30)	0.6674
Pre-gestational Diabetes	38 (1.03)	33 (1.02)	5 (1.09)	0.8916

^1^ Missing data was not included. ^2^ Age data is given as mean (SD) years. ^3^ Obesity is defined as BMI ≥ 30 kg$/m^{2}$.

**Table 2:** Obstetric Outcomes.

	Total ^1^	Vaccinated	Non-Vaccinated	*p*-Value
	(*n* = 3700), *n* (%)	(*n* = 3240), *n* (%)	(*n* = 460), *n* (%)	
Late Pregnancy Complications				
Gestational Diabetes	510 (13.78)	446 (13.77)	64 (13.91)	0.9315
PPROM ^2^	118 (3.19)	103 (3.18)	15 (3.26)	0.9255
Pregnancy-induced HTN ^3^	71 (1.92)	65 (2.01)	6 (1.30)	0.3045
Preeclampsia	48 (1.30)	42 (1.30)	6 (1.30)	0.9886
SGA ^4^	466 (12.59)	417 (12.87)	49 (10.65)	0.1797
Early preterm birth (GA < 34)	36 (0.97)	27 (0.83)	9 (1.96)	0.0216
Preterm birth (GA37 > )	232 (6.27)	197 (6.08)	35 (7.61)	0.2057
GA at delivery ^5^		38.7 ± 1.5	38.8 ± 2.0	0.0604
Mode of delivery				0.1775
Vaginal	2668 (72.38)	2351 (72.83)	317 (69.21)	
Non-urgent CS ^6^ delivery	583 (15.82)	507 (15.71)	76 (16.59)	
Urgent CS delivery	435 (11.80)	370 (11.46)	65 (14.19)	
Delivery Complications				
Maternal fever > 38	67 (1.81)	57 (1.76)	10 (2.17)	0.5325
Meconium-stained amniotic fluid	512 (14.53)	424 (13.75)	88 (20.05)	0.0005

^1^ Missing data were not included. ^2^ Premature Pre-labor Rupture Of Membranes. ^3^ Hypertension. ^4^ Small for Gestational Age. ^5^ Gestational age at delivery is given as mean ± SD, weeks. ^6^ Cesarean Section.

**Table 3:** Neonatal Outcomes.

	Total ^1^	Vaccinated	Non-Vaccinated	*p*-Value
	(*n* = 3700), *n* (%)	(*n* = 3240), *n* (%)	(*n* = 460), *n* (%)	
Birth Weight ^2^		3225.5 ± 469.4	3247.2 ± 542.6	0.1078
Infant Complications				
Apgar < 7 at 5 min	11 (0.30)	10 (0.31)	1 (0.22)	0.7366
Venous cord blood pH < 7	9 (0.24)	6 (0.19)	3 (0.65)	0.0571
Respiratory Complications	75 (2.03)	63 (1.94)	12 (2.61)	0.3441
Respiratory distress syndrome	20 (0.54)	17 (0.52)	3 (0.65)	0.7303
Transient tachypnea of the newborn	48 (1.30)	40 (1.23)	8 (1.74)	0.3708
Meconium aspiration syndrome	9 (0.24)	8 (0.25)	1 (0.22)	1.0000
Other Complications				
Mechanical ventilation	33 (0.89)	27 (0.83)	6 (1.30)	0.2905
NICU ^3^ hospitalization	105 (2.84)	89 (2.75)	16 (3.48)	0.3767
Intracranial hemorrhage	2 (0.05)	1 (0.03)	1 (0.22)	0.2332
Hypothermia	1 (0.03)	1 (0.03)	0 (0)	1.0000
Hypoglycemia	185 (5.00)	159 (4.91)	26 (5.65)	0.4928

^1^ Missing data was not included. ^2^ Birth weight is given as grams, mean ± SD. ^3^ Neonatal intensive care unit.

**Table 4:** Obstetric and neonatal outcomes multivariable models.

	Adjusted Odds Ratio ^1^ (Vaccinated vs. Non-Vaccinated); 95% CI
Late Pregnancy Complications	
Hypertensive disorders of pregnancy ^2^	1.30; 0.70–2.42
Preterm birth (GA < 37)	0.94; 0.62–1.44
SGA ^3^	1.01; 0.66–1.55
Meconium-stained amniotic fluid	0.63; 0.49–0.83
Mode of Delivery	
Non-urgent CS ^4^ vs. Vaginal	0.86; 0.64–1.14
Urgent CS vs. Vaginal	0.78; 0.57–1.07
Neonatal Outcomes	
Respiratory complications ^5^	0.74; 0.38–1.45
NICU ^6^ hospitalization	1.06; 0.54–2.1

^1^ Adjusted for maternal age, parity, smoking, gestational age at delivery and background conditions such as obesity, hypertensive disorders, and diabetes. Hypertensive disorders of pregnancy was adjusted for pregestational diabetes and chronic hypertensive disorder only. ^2^ Hypertensive disorders of pregnancy include pregnancy-induced hypertension, preeclampsia, and super imposed preeclampsia. Neonatal intensive care unit. ^3^ Small for Gestational Age. ^4^ Cesarean Section. ^5^ Respiratory complications include respiratory distress syndrome (RDS), transient tachypnea of neonate (TTN) and meconium aspiration syndrome (MAS). ^6^ Neonatal intensive care unit.

## Data Availability

Not applicable.
